# Angiomyofibroblastoma of the spermatic cord: a case report

**DOI:** 10.1186/1752-1947-4-79

**Published:** 2010-03-04

**Authors:** Nikolaos E Tzanakis, George A Giannopoulos, Stamatis P Efstathiou, Georgios E Rallis, Nikolaos I Nikiteas

**Affiliations:** 1 4th Surgical Department, Attikon Hospital, University of Athens, (1 Rimini str), Athens, (124 62), Greece; 2 Department of Internal Medicine, Hygeias Melathron Hospital, (6 Therianou str), Athens, (114 73), Greece; 3 2nd Propedeutic Department of Surgery, Laikon Hospital, University of Athens, (17 Agiou Thoma str), Athens, (115 27), Greece

## Abstract

**Introduction:**

Angiomyofibroblastoma is a benign soft tissue tumor with tendency to arise in the vulva.

**Case presentation:**

We report a 36-year-old Greek Caucasian man presenting with a left inguinal painless mass. This is the second case of angiomyofibroblastoma of the spermatic cord. At operation, a 4.5 cm well-circumscribed solid tumor was found adherent to the spermatic cord. The tumor consisted of spindle-shaped cells proliferating in short fascicles between numerous medium-sized blood vessels with thin and hyalinized walls. Neoplastic cells had eosinophilic cytoplasm with neither mitotic figures nor nuclear atypia. The stroma included abundant mast cells and few mature lypocytes. Immunostaining showed positivity for vimentin, CD34, desmin and smooth muscle actin. Our patient was treated by simple excision and was followed up for five years with clinical examination and ultrasonography, revealing no evidence of local recurrence or metastasis.

**Conclusion:**

This unusual neoplasm should be distinguished from aggressive angiomyxoma and other myxoid malignant tumors with widespread metastatic potential.

## Introduction

In 1992, Fletcher *et al*. [1] described 10 cases of a previously unrecognized benign soft tissue tumor of the vulva that was often misdiagnosed as aggressive angiomyxoma. The term angiomyofibroblastoma (AMF) was endorsed for this novel tumor. The morphologic hallmarks of this tumor were its well-circumscribed margins, prominent vascularity and features suggestive of myofibroblastic differentiation [1]. Since the aforementioned original study, there have been several additional reports of AMF of the genital tracts of both men [2,3] and women [4,5], but only a single case of this tumor arising from the spermatic cord [6]. The purpose of this study is to expand the experience with AMF by describing the second case of the latter unusual location of this rare lesion and providing a long period of follow-up.

## Case presentation

**Figure 1:**
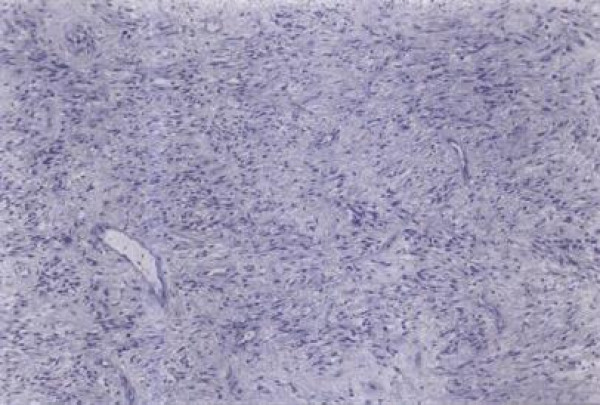
**Moderately cellular area with spindle-shaped tumor cells arranged in short fascicles between numerous vessels with collagenized walls (Hematoxylin-Eosin × 100)**.

**Figure 2:**
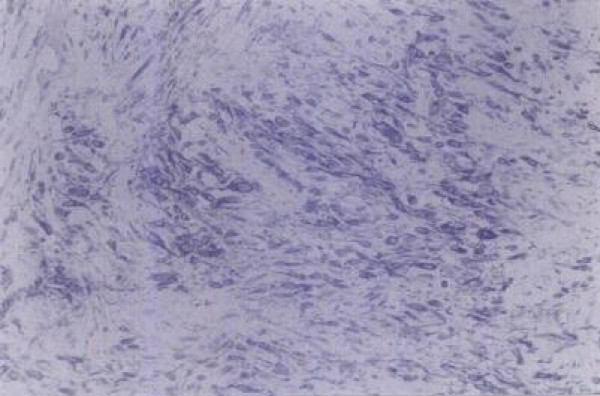
**Tumor cells with myofibroblastic morphology intensely positive for desmin (Avidin-Streptavidin method × 200)**.

A 36-year-old Greek Caucasian man presented with a left inguinal painless mass that had been growing slowly for six months. During operation, a 4.5 cm well-circumscribed solid tumor was found adherent to the spermatic cord. The testis and the epididymis were not involved. The lesion was pale gray with a vague lobular and focally glistening cut surface. On microscopic examination, the tumor was well-demarcated and consisted of spindle-shaped cells proliferating in short fascicles between numerous medium-sized blood vessels with thin and hyalinized walls (Figure 1). Focally, the tumor cells had an epithelioid appearance with eosinophilic cytoplasm, plump nuclei and neither mitotic figures nor nuclear atypia. The stroma included abundant mast cells and few mature lypocytes. Immunostaining of neoplastic cells showed intense positivity for vimentin, CD34 and desmin (Figure 2), mild positivity for smooth muscle actin and no staining for keratin and S100 protein. Our patient was treated by simple excision and was followed up for five years with clinical examination and ultrasonography of the inguinal region revealing no evidence of local recurrence or metastasis.

## Discussion

AMF is a recently-described soft tissue tumor characterized by unique morphologic features, a tendency to emanate from the vulva, and a benign biologic behaviour [1,2]. There are few reported cases of occurrence in men. The tumors were localized in the scrotum and the inguinal area of the male patients [4,5,7].

The cell of origin of AMF among males has not been identified with certainty. Regarding the female patients, it is believed that the tumor is derived from mesenchymal cells in the subepithelial myxoid stromal zone that extends from the endocervix to the vulva [2], this hypothesis explaining to some extent the propensity of this tumor to arise in the lower genital tract. All the published studies of patients with AMF have presented benign tumors, without local recurrence or metastatic potential. However, the latter possibilities cannot be excluded, since the respective follow up periods were relatively small. The above limitation is avoided in the present study, which is the first reporting a sufficiently long follow-up of five years.

The most crucial issue is to determine whether this case should be assigned to AMF or to aggressive angiomyxoma (AAM). The latter is a histologically benign soft tissue tumor, associated with a high risk of local recurrence as well as with local infiltration that often results in entrapment of nerves and mucosal glands, thus making complete excision difficult [8]. It has been suggested that AMF and AAM are related neoplasms, both included in a wide spectrum of angiomyxoid tumors, which exhibit some overlapping features and various combinations of myofibroblastic, fibroblastic and lipomatous differentiation [9]. The macroscopic characteristics of our case report are in agreement with those demonstrated by AMF rather than AAM, since the tumor was a well-circumscribed, relatively small-sized lesion with no infiltrating margins [4]. Furthermore, the recognition of cytologic features reminiscent of myofibroblastic differentiation is paramount for the diagnosis of genital AMF and its differentiation from AAM in the particular patient, whereas intralesional fat tissue as observed in our case is also more frequently found in AMF [4].

Nevertheless, although desmin expression was previously thought to be specific of AMF, this protein is no longer considered as a reliable marker for distinguishing the latter from AAM, inasmuch as immunopositivity for desmin and muscle-specific actin has more recently been shown in a substantial proportion of AAM [9]. Regarding the surgical management of these rare neoplasms (AMF and AAM), the most important factor for prognosis is the surgical and macroscopic delimitation of the tumor. Because most AMFs have been successfully treated with simple excision, this seems to be the appropriate therapy for these tumors [1-6]. After histological examination, wide excision is meanwhile required in cases of AAM because of the propensity of the latter for local recurrence [10].

The differential diagnosis of AMF also includes smooth muscle tumors, peripheral nerve sheath tumors, glomus tumor, chondroid syringoma, myxoid malignant fibrous histiocytoma, angiomyolipoma, spindle cell lipoma and myxoid liposarcoma. The distinction between AMF and these tumors has been described in detail elsewhere [1]. As most of these diagnoses were introduced before the original description of AMF, the aforementioned entities should be easily discriminated from AMF by routine light microscopic examination in conjunction with immunohistochemical studies and electron microscopic examination in selected cases [2-5]. Lastly, the staining of tumor cells in our case with antibodies to CD34, a 115-kDa transmembrane glycoprotein associated with cellular interaction and adhesion, is an additional finding compatible with the diagnosis of AMF [2-5].

## Conclusion

Although the exact nosologic position of AMF is still surrounded by some controversy and requires further elucidation, we conclude that our case represents the second report of AMF of the spermatic cord, based on its conventional histopathologic and immunophenotypic features. Simple excision appears sufficient for the surgical management of AMF, whereas wide excision after histological examination is needed for the management of the related AAM, which is associated with a high risk of local recurrence and infiltration.

## Consent

Written informed consent was obtained from our patient for publication of this case report and accompanying images. A copy of the written consent is available for review by the Editor-in-Chief of this journal.

## Competing interests

The authors declare that they have no competing interests.

## Authors' contributions

NT analyzed and interpreted our patient data, GG, SE and GR contributed equally in designing and writing the paper with NT. Meanwhile, NN was involved in drafting the manuscript and revising it critically. All authors read and approved the final manuscript.
